# Artery targeted photothrombosis widens the vascular penumbra, instigates peri-infarct neovascularization and models forelimb impairments

**DOI:** 10.1038/s41598-019-39092-7

**Published:** 2019-02-20

**Authors:** Taylor A. Clark, Colin Sullender, Shams M. Kazmi, Brittany L. Speetles, Michael R. Williamson, Daniella M. Palmberg, Andrew K. Dunn, Theresa A. Jones

**Affiliations:** 1 Institute for Neuroscience, University of Texas at Austin, Austin, Texas 78712 USA; 2 Department of Biomedical Engineering, University of Texas at Austin, Austin, Texas 78712 USA; 3 Department of Mechanical Engineering, University of Texas at Austin, Austin, Texas 78712 USA; 4 Department of Public Health, University of Texas at Austin, Austin, Texas 78712 USA

## Abstract

The photothrombotic stroke model generates localized and reproducible ischemic infarcts that are useful for studying recovery mechanisms, but its failure to produce a substantial ischemic penumbra weakens its resemblance to human stroke. We examined whether a modification of this approach, confining photodamage to arteries on the cortical surface (artery-targeted photothrombosis), could better reproduce aspects of the penumbra. Following artery-targeted or traditional photothrombosis to the motor cortex of mice, post-ischemic cerebral blood flow was measured using multi-exposure speckle imaging at 6, 48, and 120 h post-occlusion. Artery-targeted photothrombosis produced a more graded penumbra at 48 and 120 h. The density of isolectin B4^+^ vessels in peri-infarct cortex was similarly increased after both types of infarcts compared to sham at 2 weeks. These results indicate that both models instigated post-ischemic vascular structural changes. Finally, we determined whether the strength of the traditional photothrombotic approach for modeling upper-extremity motor impairments extends to the artery-targeted approach. In adult mice that were proficient in a skilled reaching task, small motor-cortical infarcts impaired skilled-reaching performance for up to 10 days. These results support that artery-targeted photothrombosis widens the penumbra while maintaining the ability to create localized infarcts useful for modeling post-stroke impairments.

## Introduction

The ischemic penumbra was first defined by Astrup and colleagues^1,2^ based on thresholds of reduced cerebral blood flow (CBF) that were associated with electrical silence (loss of somatosensory evoked responses) in cortical tissue surrounding a core region of ischemia. Astrup *et al*. demonstrated that as CBF was steadily reduced to around 40% of baseline levels, electrical dysfunction was evident, at 30% electrical failure became complete, and at 10% increased extracellular potassium and subsequent cell death occurred. Importantly, however, prompt reperfusion of CBF could salvage tissue so long as flow rates did not fall below the threshold of energy and ion pump failure. Today the penumbra is more broadly defined as a region of ischemic tissue which is functionally impaired and at risk of infarction, but has the potential to be salvaged depending on post-ischemic vascular events^3–6^. Adequate reperfusion in the penumbra early after ischemia onset limits the extent of tissue damage, and the penumbra has therefore been a focus of acute neuroprotective treatments^3,7–11^. Adequate cerebral blood flow (CBF) to the penumbra also supports cellular mechanisms of repair and remodeling over longer time periods, including neuronal growth and neovascularization, which are related to improved functional outcomes^12–23^.

The photothrombotic stroke model is well suited for the study of cellular mechanisms of functional recovery following ischemia because it creates circumscribed lesions that can be placed with anatomical precision, for example, in discrete functional regions of cortex to reliably cause behavioral impairments^24–26^. One drawback of the model is that the ischemic penumbra is narrow, making it challenging to examine how events unfolding within the penumbra relate to the repair and remodeling responses that support improved functional outcomes^24^.

During photothrombosis, following administration of a photoactive dye (Rose Bengal), illumination of a circumscribed region generates singlet oxygen, platelet activation, and damage to endothelial cells^25,26^. This results in vasogenic edema, which rapidly propagates the developing lesion beyond the illuminated region, and which is thought to compromise the development of a penumbra^25,26^. Furthermore, within the circumscribed region of illumination there is non-discriminant tissue damage inclusive of microvasculature and veins. We recently established a variation of the photothrombotic approach, artery-targeted photothrombosis, in which a digital micromirror device (DMD) is used to confine illumination to a set of pre-identified arterial branches on the cortical surface, thereby minimizing damage to surrounding brain tissue^27^. We then used this method to simultaneously monitor oxygen tension and CBF after vascular occlusion. However, our previous study did not directly compare the artery-targeted to the traditional photothrombotic approach. The main goal of the present study was to test whether this modified photothrombotic stroke model could better reproduce a graded vascular penumbra compared to the traditional model.

Another goal was to determine whether there are changes in vascular density in peri-infarct cortex that may reflect neovascularization. The magnitude and persistence of neovascularization in peri-infarct tissue^22^, and its significance for chronic post-stroke outcomes, is unclear. In rodent models of focal ischemia^12–18^, and after stroke in humans^19–21^, increased vascular density in the peri-infarct cortex has been associated with improved outcomes, but considerable variability in the timing and extent of neovascularization has been reported across studies. Additionally, despite ample histological evidence for neovascularization in other focal ischemia models^12–18,28–35^, there is only sparse evidence for it in the traditional photothrombotic model^36^. Recent i*n vivo* imaging studies of perfused vessels revealed no evidence for neovascularization after photothrombosis^37–39^, but this visualization approach cannot rule out the existence of newly formed vessels that are not yet perfused. Here, we used histological measures of lectin-labeled vessels to assess vascular density change and its potential spatiotemporal variation after artery-targeted and traditional photothrombosis.

A final goal was to determine whether the new model maintains the strength of the traditional one for the study of recovery mechanisms. Sufficient damage to the caudal forelimb area of motor cortex results in deficits in skilled forelimb reaching tasks that have been used to model chronic post-stroke upper-extremity impairments^40–46^. Photothrombotic infarcts of the caudal forelimb area reliably impair skilled reaching performance in mice^47–50^ and thus have been a popular choice for modeling upper extremity impairments in mice. Here we tested whether artery-targeted photothrombosis maintains the strength of the traditional photothrombosis approach for modeling upper extremity impairments in mice.

## Materials and Methods

### Subjects

A total of 44 well-handled young adult (4–6 mo) C57/Bl6/YFP-H line mice of both sexes were used to examine the impact of artery-targeted photothrombosis in motor cortex (MC) on CBF and vascular density (*n* = 21) and skilled forelimb function (*n* = 23). Estimations of sample sizes for CBF and vascular density measures were based on previous research examining post-ischemic CBF^37^, as well as calculations of the minimum sample size needed to obtain a significant result with a power level of 0.8, from preliminary pilot data. Equal numbers of male and female mice were randomly placed into one of three groups prior to any experimental procedures: artery-targeted photothrombosis (targeted: *n* = 5 male, *n* = 4 female), traditional photothrombosis (traditional: *n* = 4 male and *n* = 4 female) or sham (*n* = 2 male, *n* = 2 female). One male in the targeted group, and 2 females in the traditional group were included in vascular density, but not CBF measures due to poor window clarity, and 1 male in the traditional group was included in CBF, but not vascular density measures due to uneven lectin labeling across hemispheres. For a comprehensive breakdown of animal numbers by group refer to Table 1. Not included in the n’s above was 1 animal in the traditional group that was omitted from CBF and vascular density measures due to a lesion volume more than two standard deviations above the group mean, a criterion defined prior to experimental procedures.

The impact of artery-targeted photothrombosis of MC on skilled forelimb function was tested in young-adults undergoing targeted-photothrombosis (*n* = 7 male, *n* = 6 female) or sham procedures (*n* = 6 male, *n* = 4 female). Not included in the n’s above were 2 males and 1 female omitted from the behavioral measures due to lesion volumes two standard deviations smaller than the group average, and 1 male that failed to learn the task.

As reported in Supplementary Materials, we additionally performed targeted photothrombotic infarcts in middle-aged mice to probe the influence of age on CBF reductions and vascular density changes.

Animals were bred at the Animal Resource Center at the University of Texas at Austin. Mice were housed in groups of two to four on a 12:12 hour light/dark cycle. Each cage was supplied with wooden toys, bedding and PVC pipes (cage enclosures; i.e. mice nest within them) that were replaced weekly. Animals used for CBF and vascular density measures were given food and water *ad libidum*. Mice used in the behavioral study were placed on scheduled feeding (2.5–3 g food/day) for the duration of shaping, training and testing procedures. The *M* ± *SD* starting weights were 20.97 ± 0.1.04 g for females and 24.69 ± 2.3 g for males. Animals were weighed daily for the first week and then once a week to ensure they did not fall below 90% of their free-feeding body weight. Animal use was in accordance with IACUC guidelines (AUP-2015-00182) approved by the Animal Care and Use Committee of the University of Texas at Austin.

### Cranial Window Creation

Mice were anesthetized with ketamine (4 mg/kg, i.p.) and xylazine (3 mg/kg, i.p.). Dexamethasone (2 mg/kg s.c.) and carprofen (2.5 mg/kg, s.c.) were administered pre-operatively to help minimize cortical swelling and inflammation during the procedures. Anesthetic plane was monitored via respiratory rate and toe pinch response throughout surgery. Booster injections of ketamine (4 mg/kg) were given as needed to maintain anesthesia. Following a midline incision of the scalp, a 3 mm circular region of skull over frontoparietal cortex was thinned using a high-speed dental drill and 0.5 mm diameter drill bit (Fine Science Tools, product #19007-05) and removed, leaving dura intact. Saline was frequently applied to protect the brain from overheating. Skull was replaced with a 3 mm diameter No. 1 coverglass (Warner Instruments, #64-0720) and sealed with cyanoacrylate (3M Vetbond Tissue Adhesive, #1469) and dental cement. All windows were made over the forelimb area of the MC, defined from previous intracortical mapping experiments (Tennant *et al*., 2011). Medial and anterior edges of the window were approximately 2 mm rostral to bregma and 0.5 mm lateral to midline. For animals undergoing behavioral procedures, windows were placed over MC contralateral to the preferred-for-reaching forelimb. Following surgery, animals were given buprenorphine (3 ml/kg, s.c.) for pain and allowed to recover in their cage. For the first week after surgery, animals were given carprofen daily (2.5 mg/kg, i.p.) to minimize inflammation that contributes to window clouding.

### Artery-targeted and Traditional Photothrombosis

**Figure 1:**
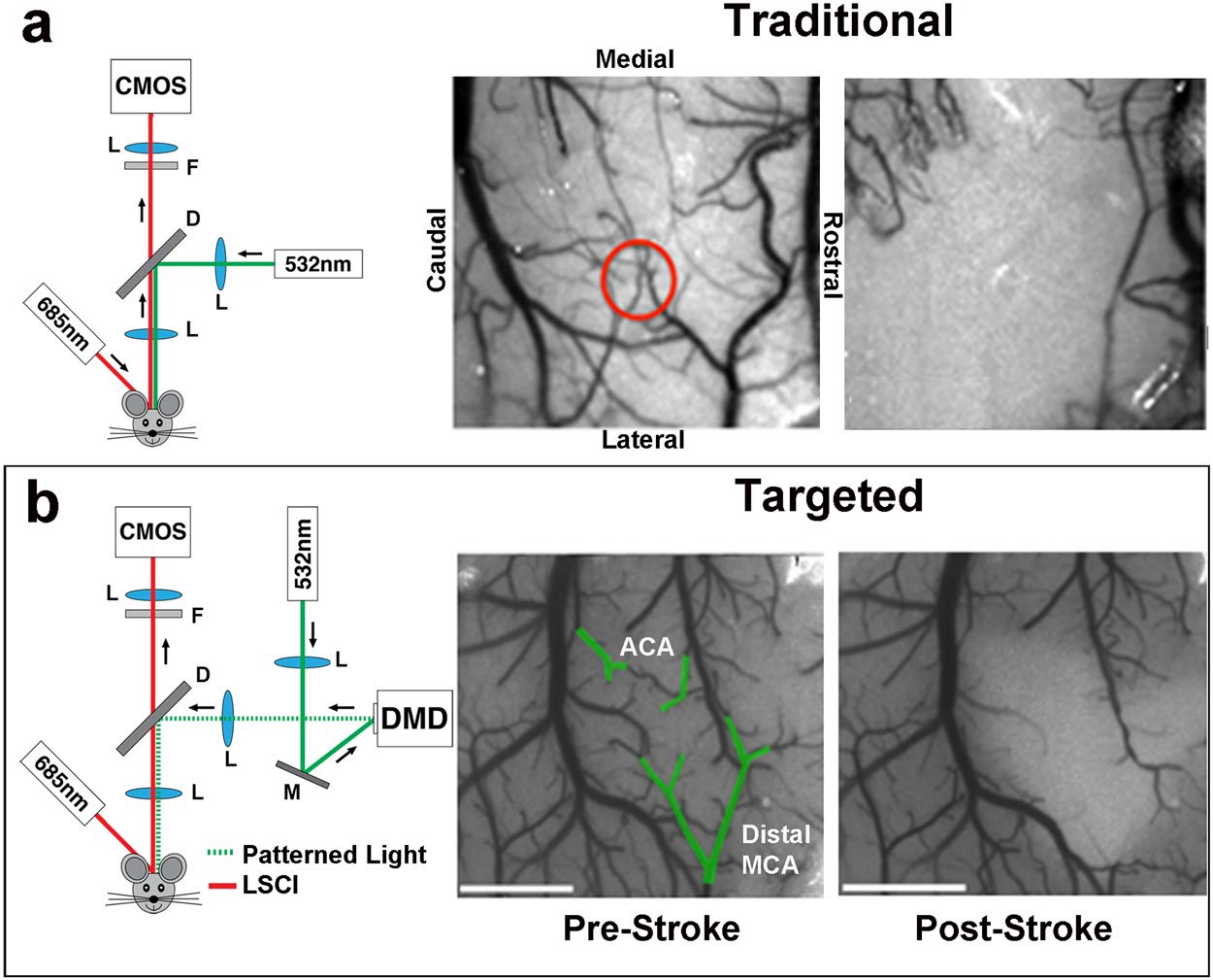
Overview of artery-targeted and traditional photothrombosis induction methods. Schematic diagrams of (**a**) traditional and (**b**) artery-targeted photothrombotic setups, and laser speckle contrast images (LSCI) depicting CBF before (left) and 30 minutes after (right) each. All images are shown in the same orientation. For traditional photothrombosis, a 532 nm laser was passed through a dichroic mirror to deliver focused photothrombosis over a cortical region (red circle). For artery-targeted photothrombosis, the 532 nm laser was coupled with the DMD (digital micromirror device) to provide patterned illumination over the cranial window (green outlines). The distance between arteries did not impact the area of ischemic core/penumbra. On both setups, a separate 685 nm laser provided oblique illumination for LSCI. CMOS, complementary metal oxide semiconductor. L, lens; M, mirror; F, filter. ACA, anterior cerebral artery; MCA, middle cerebral artery.

Mice were anesthetized with isoflurane (4% induction, 1.5–2% maintenance) in O_2_ and affixed to a stereotaxic frame. Arterial oxygen saturation and heart rate from pulse oximetry (MouseOx; Starr Life Sciences Corp. Oakmont, PA, USA) were recorded and temperature was maintained at 37 °C with a feedback temperature control system (FHC Bowdoin, ME, USA). For targeted photothrombosis, a green diode laser (532 nm, Millenia V, Spectra Physics, Santa Clara, CA, USA) was coupled to a digital micro-mirror device (DMD, LDD400-1P, Wavelength Electronics, Bozeman, MT, USA) to provide patterned illumination (20 mW) over arteries on the pial surface supplying MC, with minimal exposure to surrounding parenchyma (Fig. 1a). In order to account for variations in the size and caliber of the arteries targeted, a range of 1–3 branches were illuminated. The *M* ± *SD* target area was 0.15 ± 0.098 mm^2^. The main targets were distal branches of the middle cerebral artery (MCA). In animals with extensive collateralization (n = 4), 1–2 distal branches of the anterior cerebral artery (ACA) were also illuminated to control collateral flow at the time of occlusion. Thirty seconds following retroorbital injections of Rose Bengal (50 µL, 15 mg/mL i.v., Sigma), targeted vessels were irradiated with the patterned laser. For traditional photothrombosis, following the injection of Rose Bengal (100 µl, 15 mg/ml i.p.), a green laser fiber coupled with a separate objective system (10×, Olympus) delivered focused laser illumination (20–24 mW) over a defined region of MC for 12–15 minutes (Fig. 1b). Sham animals were exposed to laser illumination after injections of sterile saline. CBF was monitored in real time throughout and 30 min following the occlusion using traditional laser speckle contrast imaging^51–53^. The backscattered laser light was relayed to a CMOS camera (acA1300–60gmNIR, 1280 × 1024 pixels, Basler AG, Germany) with ∼2x magnification and acquired at 60 frames-per-second with 5 ms exposure time using custom software.

### Multi-Exposure Speckle Imaging of Penumbral CBF

CBF was monitored following targeted or traditional photothrombosis using multi-exposure speckle imaging (MESI) as previously described^51–53^. Briefly, a laser diode (660 nm, Micro Laser Systems, Garden Grove, CA, USA) illuminated the craniotomy while simultaneously triggering 15 camera (A602f; Basler Vision Technologies, Ahrensburg, Germany) exposure durations ranging from 0.05 to 80 ms. Backscattered light was collected by a 5x objective and imaged onto the camera. A 7 by 7 pixel window was used to convert the raw frames to speckle contrast images^51,52^. For all MESI imaging sessions, mice were anesthetized and affixed to a stereotaxic frame, as described above. Within animals, heart rate was matched within 10% between baseline and post-infarct imaging sessions by controlling the depth of anesthesia and therefore regulating cardiac output. Post occlusion MESI imaging sessions occurred at 6, 48, and 120 h post stroke. Data from 3 animals at 6 h (*n* = 2 targeted, *n* = 1 targeted MA) and from 2 animals at 120 h (*n* = 1 traditional, *n* = 1 targeted MA) had to be excluded due to an inability to sufficiently match heart rate to baseline conditions. Data from these mice were included in all other time points.

**Figure 2:**
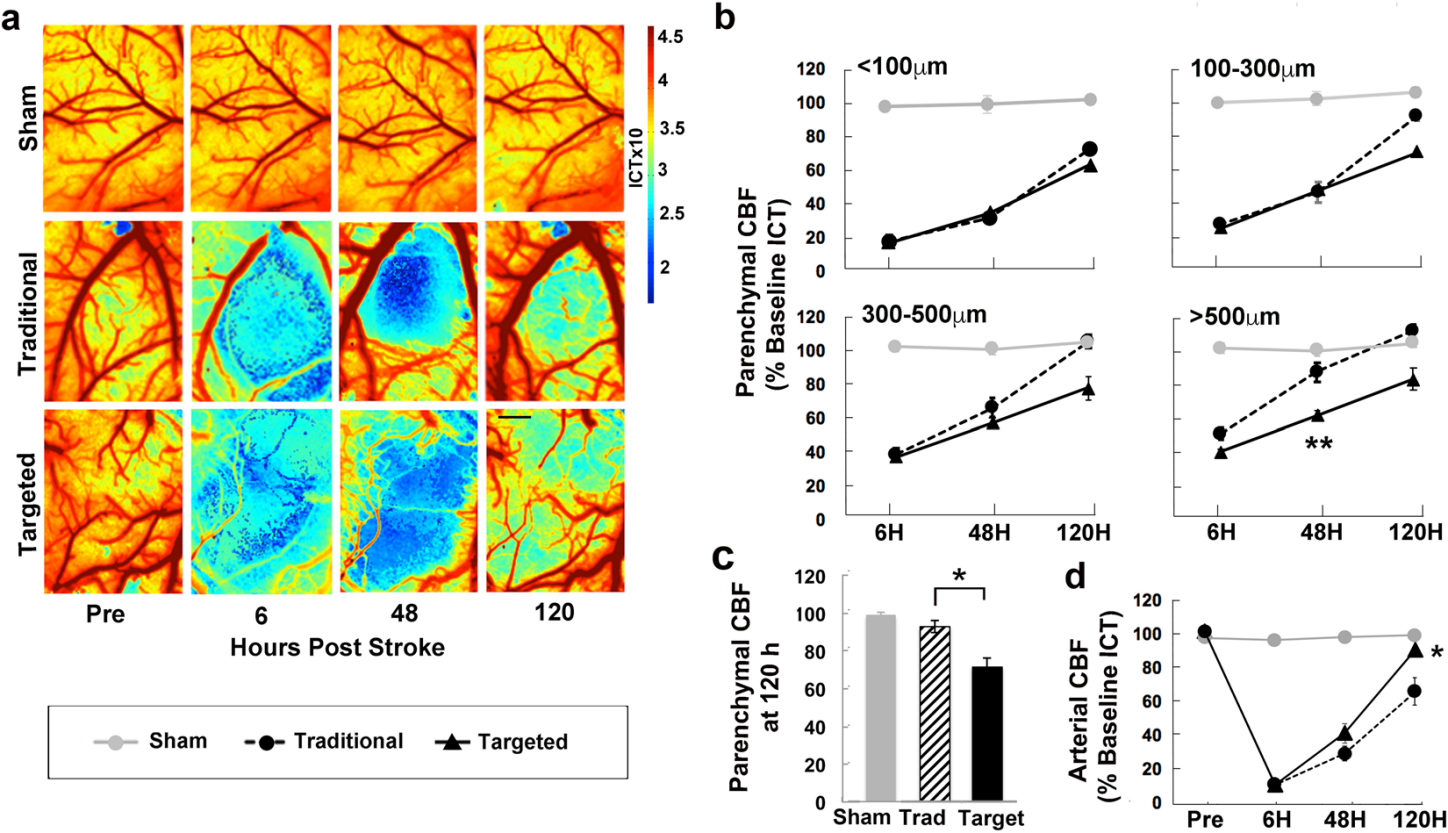
Artery-targeted photothrombosis increased the area of reduced CBF in surrounding cortical tissue. (**a**) Representative inverse correlation time (ICT) images acquired using multi-exposure speckle imaging (MESI). Areas of higher CBF are represented in red, and areas of lower CBF are represented in blue. Scale bar = 500 µm. (**b**) Post-ischemic CBF deficits at each imaging time point represented as a % of baseline ICT values at different distances. At distances >500 µm, CBF was significantly increased in the traditional group compared to the targeted group at 48 h. (**c**) Parenchymal CBF pooled across distances at 120 h post infarct was significantly greater in the traditional group compared to the targeted group. (**d**) CBF in occluded arteries was significantly higher at 120 h in the targeted compared to traditional group. **p < 0.001, *p < 0.02.

Raw images captured by the camera were converted to speckle contrast images as a ratio of the *SD* to *M* intensity of individual pixel values within a small region of the image^51,53^. The speckle contrast image was then calculated using a 7 by 7 pixel sliding window, and was computed, displayed, and saved in real-time using an efficient processing algorithm^51,53^. After processing, speckle contrast images were averaged and converted to inverse correlation time (1/τc, ICT) images (Fig. 2) providing a more quantitative measure of CBF^38,53^. The ischemic core was defined at 48 h as the area of parenchyma with CBF values ≤20% of baseline, a cutoff that was supported by comparisons between CBF reductions and histological damage explained below. The use of the 48 h time point to estimate core size *in vivo* was intended to help ensure that the transition of ischemic tissue to infarct core fate would be complete. For core measurements, ICT image sequences at 48 h were thresholded against baseline ICT image sequences, vessels extracted, and matched by heart rate. Ten to twelve individual CBF measurements were then gathered from raw MESI data using a custom-made MATLAB script that extracted ICT from speckle contrast images. Measurements were binned according to distance from the ischemic core (<100, 100–300, 300–500 and >500 µm) and then averaged. The furthest distance (>500 µm) ranged between 700 µm and 1 mm between animals depending on the size and location of the core in relation to the edge of the cranial window. Analysis of penumbral CBF was performed blind with respect to infarct group. Results are presented as % baseline CBF.

### Tissue Processing and Analysis of Lesion Volume

**Figure 3:**
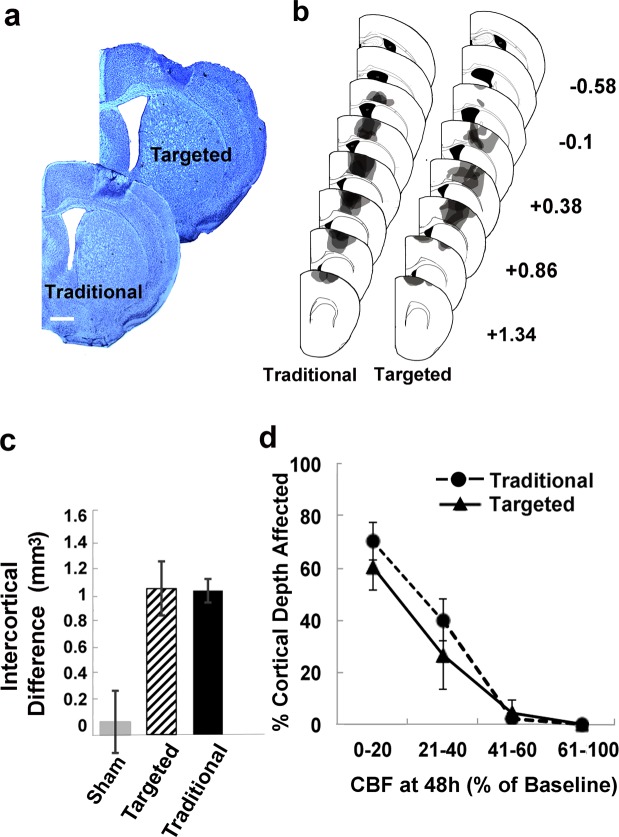
Lesion depth in histological tissue sections corresponded to cortical areas with the greatest CBF reductions following artery-targeted and traditional photothrombosis. (**a**) Representative Nissl stained coronal sections from each infarct group. Sections are approximately 0.14 mm anterior to bregma. Scale bar = 500 µm. (**b**) Reconstructions of each infarct overlayed on coronal section templates. Numbers to the right are anterior to posterior coordinates (mm) relative to bregma. (**c**) Infarct size, as estimated by the difference between contralesional and peri-infarct cortical volumes, was similar between infarct groups. (**d**) Relationship between CBF deficits assessed with MESI at 48 h and cortical damage (lesion depth). In both targeted and traditional groups, the greatest cortical damage corresponded to areas where CBF fell to or below 20% of baseline CBF at 48 h. Areas where CBF was at or above 40% of baseline CBF showed little to no cortical damage in histological sections.

Animals were overdosed with sodium pentobarbital and transcardially perfused with 0.1 M phosphate buffer saline (PBS) and 4% paraformaldehyde. For CBF and vascular density measures, animals were euthanized at either 7 (*n* = 4 targeted, *n* = 4 traditional) or 14 days (*n* = 5 targeted, *n* = 4 traditional, Fig. 3) after photothrombosis or sham procedures. For behavioral measures, animals were euthanized 30 days after photothrombosis. Following perfusion, brains were extracted and stored in 4% paraformaldehyde for <48 h before being sliced into 40 µm thick coronal sections using a Leica VT1000S vibratome. Every sixth section was mounted onto gelatin-coated slides and Nissl stained with toluidine blue. Contralesional and remaining ipsilesional cortical areas were measured in eight coronal sections per animal between approximately 1.34 mm anterior to 0.58 mm posterior to bregma spaced 240 µm apart with Neurolucida^TM^ software at a final magnification of 17x. Cortical measurements were performed by an experimenter (author D.P.) that was blind to experimental conditions. Cortical volume was estimated as the product of summed section areas and the distance between sections. Lesion volume was then calculated as the difference between volumes of the contralesional and ipsilesional cortices^54^.

### Comparison of MESI-estimated core sizes and histological infarct volume

MESI has been shown to accurately quantify perfusion boundaries in ischemic and non-ischemic tissue regions following photothrombosis^51,53^, and to provide reliable spatial perfusion indices that help to characterize vascular progression after ischemic stroke^38^. Because CBF is estimated from scattered light on the surface of the cortex, MESI is most accurate at measuring CBF in superficial cortex. We therefore sought to determine the extent to which tissue loss as measured in histological coronal sections corresponded to *in vivo* estimates of core sizes, as measured by the area in which CBF deficits were ≤20% of baseline values at 48 h after photothrombosis. Lesion depth was mapped in 2D space based on reconstructions of lesion extents on templates of coronal sections spaced 240 µm apart and estimated as a percent of cortical depth at medial to lateral increments of 25 µm. ICT images were then overlaid relative to bregma coordinates onto images of the cortical surface taken during cranial window implantation, and *M* CBF was recorded in 25 × 25 µm bins. The overlap region used for analyses fell between 1.34 and −0.1 mm anterior and posterior to bregma, and between 1 and 3 mm lateral from bregma (total area 3.36 µm^2^). The *M* percent infarct depth of cortical regions in which CBF was between 0–20, 21–40, 41–60 and 61–100% of baseline CBF was then calculated (Fig. 3d). The association between histological damage and CBF reductions at 48 h was of primary interest because this time point was used to define the infarct core using MESI, but similar assessments of the association of histological damage and CBF deficits at 6 and 120 h are presented in supplemental materials.

### Vascular labeling with isolectin B4

To visualize vasculature, free-floating sections were labeled with IB4 (Griffonia simplicifolia, 1:50, Sigma-Aldrich cat no. L2140), using a protocol adapted from Walchli *et al*.^55^. Briefly, sections were immersed in 50 mM NH_4_Cl in 0.1 M phosphate-buffered saline (PBS) for 30 minutes. After several washes with PBS, sections were incubated in 50 mM glycine in 0.1 M Tris (pH 8.0) for 5 minutes at 80 °C, and then washed in 0.1 M PBS. Sections were then permeabilized in 0.1 M Tris-buffered saline and Triton X-100 (0.3% vol/vol) for 10 minutes, and incubated for 72 h with gentle shaking on an inclined table at 4 °C in biotinylated IB4 diluted (1:50) in CaCl_2_-containing buffer (0.1 mM CaCl_2_, 0.1 mM MgCl_2_ and 0.1 mM MnCl_2_ diluted in 0.1 M PBS (pH 6.8) and blocking solution (0.05% vol/vol Triton X-100 and 2% vol/vol normal goat serum in CaCl_2_-containing buffer). Following incubation, sections were washed in PBS and incubated overnight at 4 °C in Cy3-conjugated strepdavidin (1:200, Jackson Laboratories, cat. no. 016-500-084) diluted in blocking solution. Sections were then thoroughly washed in PBS and mounted onto glass slides.

### Analysis of Vascular Density

**Figure 4:**
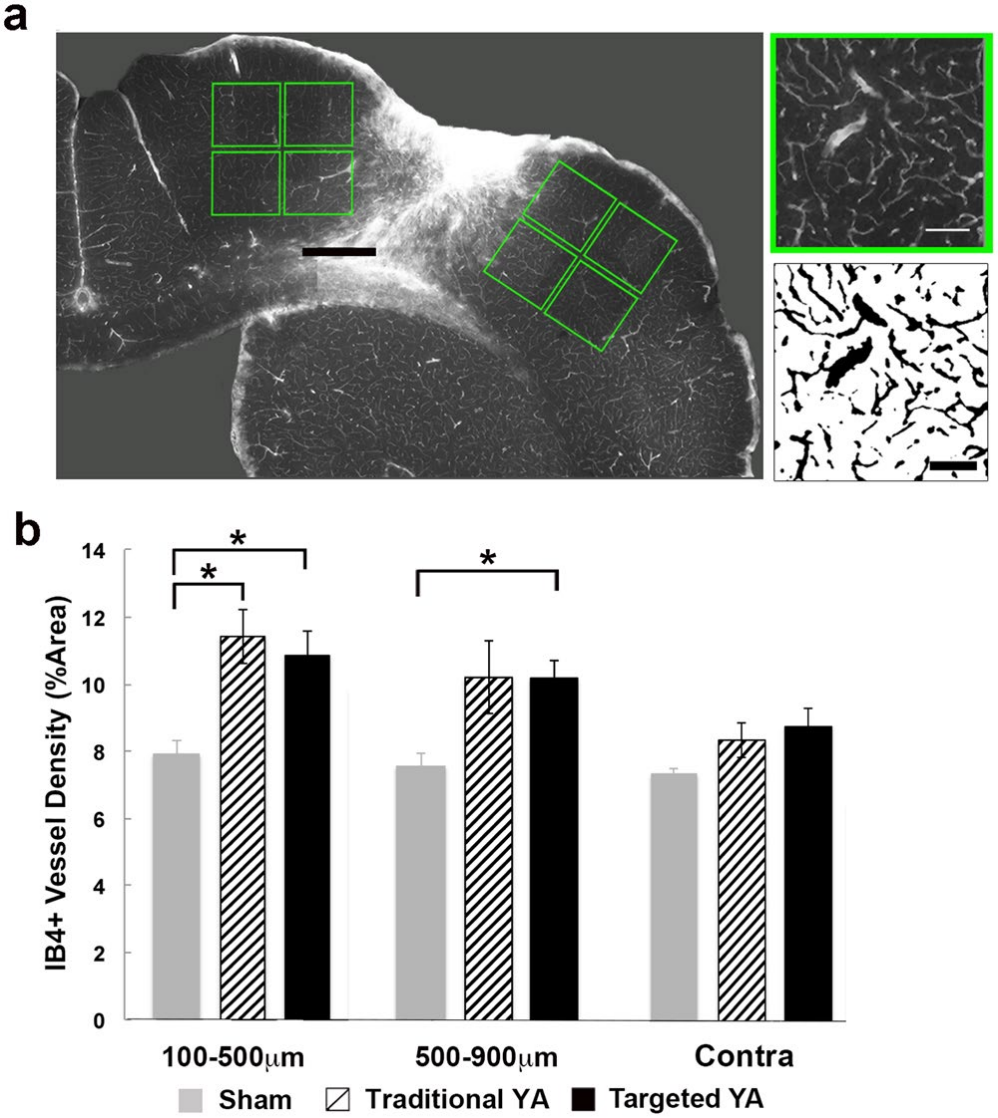
Artery-targeted and traditional photothrombosis increased vascular density in peri-infarct cortex. (**a**) Photomicrograph of IB4 labeling in the ipsilesional hemisphere at low magnification. Green insets correspond to sample sites. To the right is a higher magnification image of IB4 labeling (top right) and corresponding binarized image used for area fraction measurements (bottom left). Scale bars = 500 µm (left) and 100 µm (right). (**b**) Vascular density represented as the % area of IB4-labeled blood vessels. Compared with sham, area fractions of vessels in the ipsilesional cortex were increased in both targeted and traditional groups between 100–500 µm, and in the targeted group between 500–900 µm. Vessel densities per sample region were similar across targeted and traditional photothrombotic conditions. *p < 0.02 versus Sham.

Images of IB4-labeled tissue sections were visualized using a standard light microscope with a reflected fluorescence system (Olympus America Inc; Melville, NY) and TRITC filter. For each of 3 coronal sections per animal containing a visible lesion, eight 400 µm by 400 µm images of peri-infarct cortex were collected. Per section, a set of four images was obtained from deeper and superficial cortex within 100 µm of the medial and lateral edges of cortical infarcts, and an additional set of four was taken immediately adjacent to the first (Fig. 4). Samples in the contralesional cortex and in sham animals were taken homotopic to those collected in the ipsilesional cortex at a final magnification of 756X in ImageJ, using a custom macro that calculated the area fraction of IB4-labeled vessels in thresholded and binarized images. Analyses of vascular density were performed blind with respect to experimental group and distance from the infarct border. Vascular densities in the contralesional cortex were similar across distances, and therefore pooled across sample positions.

### Skilled Forelimb Training and Assessment

**Figure 5:**
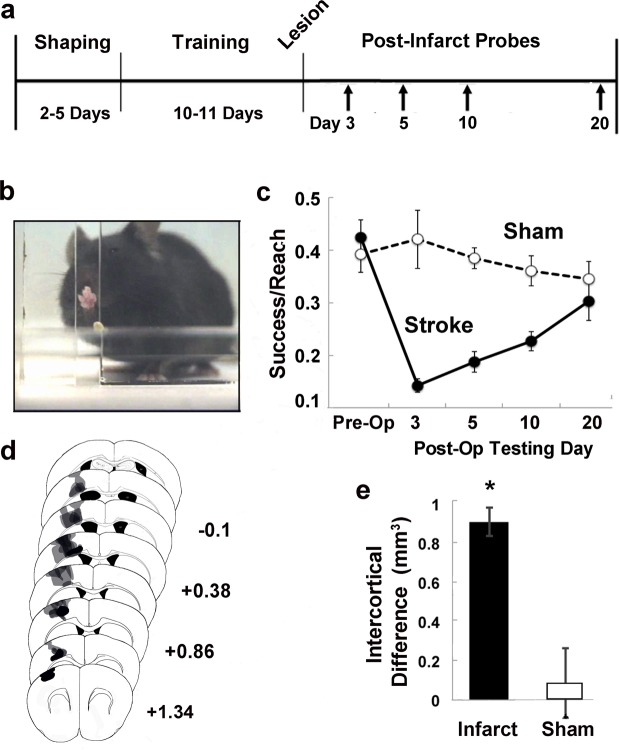
Artery-targeted photothrombotic infarcts of mouse motor cortex creates deficits in skilled forelimb reaching. (**a**) Experimental timeline for behavioral studies. (**b**) A mouse performing the single seed retrieval (SSR) task. (**c**) Pre- and post-operative reaching performance measured by the number of successfully retrieved seeds per reach attempt. Artery-targeted photothrombosis significantly impaired postoperative performance compared to shams. (**d**) Representative lesion reconstructions of the infarct group overlayed on coronal section templates. Numbers to the right represent anterior to posterior coordinates in mm relative to bregma. Infarct volume as estimated by intercortical volume difference. *p = 0.001 versus Sham.

Skilled reaching tasks are very sensitive to impairments in fine motor function of the forelimb in rodents^40–46^ and thus are often used in conjunction with traditional photothrombotic infarcts of MC to model poststroke upper extremity impairments in mice^47–50^. The impact of artery-targeted photothrombosis in MC on forelimb function was examined by testing a separate cohort of mice on performance of a skilled reaching task established prior to the infarcts^54,56^ (Fig. 5). Mice were trained to reach for a millet seed placed on a platform outside of custom-made clear Plexiglas training chamber (20 cm tall, 15 cm deep, and 8.5 cm wide, measured from outside; 0.5 cm thick Plexiglas). There were 4 mm wide vertical openings on the left and right sides of the chamber for mice to reach through with either the left or right paw. The platform (8.5 cm long, 4 cm wide, and 1.2 cm tall) contained 3 wells of varying distances for positioning seeds. Relative to the opening edge closest to the chamber center, two of the wells were positioned at distances of 3 mm (position 1) and 7 mm (position 2) away from the opening and 2 mm further from the center of the chamber. A third well (position 3) was 5 mm away and 6 mm further from the center. During initial shaping, mice were allowed to reach for millet seeds outside of both openings with either limb. The preferred-for-reaching forelimb was defined as the first limb used to make five consecutive reach attempts. For the remainder of shaping (~2–3 days), mice were encouraged to reach for a single seed placed in position 1 outside of the chamber opening corresponding to their preferred forelimb. Training started once mice were able to successfully retrieve the pellet 10 times from position 1 and lasted 10–11 days. Seeds were placed in one of the three positions per trial, with each position recurring ten times in randomized order for a total of 30 trials per day. Per trial, mice were allowed four reach attempts. A reach attempt was counted as a success when the mouse grasped the seed and brought it inside of the chamber to its mouth. Unsuccessful reach attempts included those in which the seed was missed, displaced or dropped before eating. On the last two training days prior to infarcts, the *M* ± *SD* success per attempt was 0.44 ± 0.15 (position 1), 0.30 ± 0.14 (position 2), and 0.20 ± 0.28 (position 3). Position 3 trials were omitted from analyses due to much lower preoperative performance, and much greater variability. Data were analyzed as the percent of successful reaches per attempt for positions 1 and 2. Reaching performance was tested on days 3, 5, 10 and 20 following infarct induction or sham procedures.

### Statistical Analyses

All statistical analyses were performed using the SPSS software package. Spatial differences in CBF responses to targeted and traditional photothrombosis were assessed per each post-infarct imaging time point using 2-way ANOVAs with distance from infarct core as a within-subjects variable and group (photothrombosis method) as a between-subjects variable. Each infarct group was additionally compared to the sham group in secondary analyses. When warranted by significant group-by-day interactions, post-hoc Bonferroni-Holm corrected t-tests compared groups at each distance. For CBF analyses, statistical power was 0.98 for analyses of CBF at distances greater than 500 µm at the 48-h time point, and 0.85 for pooled CBF at 120 h (See Fig. 2b,c). The same group comparisons as above in vascular density at 100–500 µm and 500–900 µm distances were assessed using independent samples t-tests. For vascular density measures, statistical power (for combined distances) was 0.98 for comparisons between the targeted and sham groups, and 0.93 for traditional and sham groups. Differences between targeted and traditional groups in the relationship between MESI estimates of CBF at the 48-h time point and structural tissue damage were tested using ANOVA with CBF as a within-subjects factor. Group differences (stroke vs. sham) in post-infarct reaching performance were examined using repeated measures ANOVAs with group as between-subjects factors, and time as a within-subjects factor. Statistical power for analyses of post-infarct behavioral test days 3,5 and 10 was above 0.9. Data from males and females were combined for the primary analyses because there were no notable differences in the patterns of experimental results between them. Supplemental materials show results disaggregated by sex. Data are reported as *M* ± *SD*.

### Compliance with Ethics Requirements

All institutional and national guidelines for the care and use of laboratory animals were followed.

## Results

### Artery-targeted photothrombosis produced a larger penumbra

Previous *in vivo* studies assessing CBF after cortical photothrombotic infarcts found a circumscribed area of reduced CBF around the ischemic core, outside of which CBF was relatively normal^37^. We measured CBF at 6, 48, and 120 h following either targeted or traditional photothrombosis at varying distances relative to the ischemic core, defined as the region with <20% of baseline CBF at 48 h. We found a main effect of distance at each imaging time point (6 h: F_[3,30]_ = 44.80, p < 0.001; 48 h: F_[3,36]_ = 68.55, p < 0.001; and 120 h: F_[3,33]_ = 17.21, p < 0.0001), confirming that CBF deficits were more severe closer to the core area of ischemia (Fig. 2b and Suppl. Table 1). At 6 h, there was no significant main effect of Group (traditional vs. targeted) or Group by Distance interaction in CBF. However, at 48 h, ANOVA revealed a significant Group by Distance interaction (F_[3,36]_ = 13.33, p < 0.0001) supporting that group differences in CBF depended on distance from the ischemic core. CBF was significantly higher in the traditional compared to targeted group at distances >500 µm from the ischemic core (t_[11]_ = 2.2, p < 0.0001, Fig. 2b). At 120 h, there was a significant main effect of Group (F_[1,11]_ = 11.25, p < 0.0001, Fig. 2c), reflecting greater CBF in the traditional compared to targeted group, an effect which did not vary significantly with distance from the ischemic core at this time point (Group by Distance interaction: F_[3,33]_ = 2.88, p = 0.051). The pattern of CBF reductions after targeted photothrombosis was not significantly different in middle aged mice compared with young adults (Suppl. Materials). Notably, in the traditional but not targeted group, CBF returned to baseline at 48 h and surpassed baseline at 120 h at distances ≥300 µm. Thus, artery-targeted photothrombosis instigated more sustained deficits in cerebral perfusion at further distances. These results are consistent with prior findings of a sharp border between ischemic and non-ischemic territory following traditional photothrombosis^37^ and provide evidence for a more graded distribution of CBF deficits in the peri-lesion cortex following artery-targeted photothrombosis.

The more sustained decrease in cortical CBF in the targeted group was despite significantly higher arterial CBF compared to the traditional group (main effect of Group: F_[1,7]_ = 25.59, p < 0.0001), an effect that varied significantly across time points (Group by Time: F_[2,14]_ = 8.98, p = 0.003). Post-hoc t-tests indicated that arterial CBF was significantly greater in the targeted compared with traditional group at 120 h, but not earlier (Fig. 2c). This suggests that there was a greater degree of delayed spontaneous reperfusion after artery-targeted photothrombosis.

### Histological damage paralleled regions of severe CBF deficits

Despite differences in post-ischemic CBF patterns, lesion volumes were comparable between targeted and traditional groups (Fig. 3a,b and Suppl. Table 2), and contralesional cortical volumes were similar to sham animals (*M* ± *SD* mm^3^, targeted: 61.7 ± 4.8, traditional: 58.5 ± 2.5, sham: 59.2 ± 2.6). Furthermore, variation in lesion volumes was small in both infarct groups (lesion volume SD, targeted: ±0.21; traditional: ±0.47).

We next assessed the spatial relationship between the MESI estimated regions of CBF reductions at 48 hand the depth of structural tissue damage in Nissl stained coronal sections at 1 to 2 weeks after photothrombosis (Fig. 3c). Across groups, structural damage in histological sections was only observed in areas where CBF was less than or equal to 40% of baseline CBF. Furthermore, areas where CBF dropped below 20% at 48 h exhibited the most severe tissue damage, supporting MESI estimates of ≤20% baseline CBF at 48 h as a reliable *in vivo* estimate of the ischemic core. The relationship between CBF deficits and cortical lesion depth did not differ significantly between infarct groups (Group by CBF reduction effect on lesion depth: F_[1,9]_ = 0.51, p = 0.49). Furthermore, the core surface area, based on MESI estimates of ≤20% baseline CBF at 48 h, was similar between groups (*M* ± *SD* core surface area in mm^2^, targeted = 0.98 ± 0.26; traditional = 1.17 ± 0.45). The average % CBF within the MESI defined core was also similar between groups. (*M* ± *SD* CBF in the MESI defined core, targeted = 16.5% ± 1.7%; traditional = 15.5% ± 2.7%).

Because CBF was measured at defined distances from the borders of the ischemic core, it was possible that these measures could vary between groups in distance from the center of the ischemic core. Given that distance from center changes in proportion with area, this was probed by calculating the ratio within animals of the mean distance of samples in the ≥500 distance bin and the square root of the estimated core surface area (i.e., Mean distance of samples in µm/square root (surface area in µm^2^). This ratio was found to be similar between infarct groups. (*M* ± *SD*, targeted = 0.52 ± 0.13*;* traditional = 0.63 ± 0.15), supporting that samples were similarly positioned across groups relative to infarct cores and borders.

### Vascular density was increased after photothrombosis

Previous *in vivo* imaging studies of filled vessels have not found evidence for neovascularization in cortex near photothrombotic infarcts^37–39^, but some studies have found histological evidence for increased vascular density in other models of focal ischemia^16–20,26–33^. Here, the density of IB4-labeled blood vessels did not vary between survival times in either group (*M* ± *SD* % area fraction, targeted at 7 d: 10.0 ± 1.0, at 14 d: 11.0 ± 1.0; traditional at 7 d: 10.6 ± 2.4, at 14 d: 11.3 ± 2.4), and therefore the data were combined across time points. Vascular density in samples between 100–500 µm from the infarct border was significantly greater after both types of photothrombosis compared to sham animals (Fig. 4; targeted: t_[10]_ = 2.23, p = 0.01; traditional: t_[10]_ = 2.23, p = 0.003; see also Suppl. Table 3). Between 500–900 µm from the infarct border, vascular density was significantly greater in the targeted group, but not traditional group, compared to sham (targeted: t_[11]_ = 2.23, p = 0.002; traditional: t_[10]_ = 2.23, p = 0.12). However, mean vascular densities between the photothrombotic groups were not different. Vascular density was similar between groups in the contralesional cortex, although there was a nonsignificant tendency for it to be greater in photothrombotic groups relative to sham (targeted vs. sham: t_[10]_ = 2.20, p = 0.06; traditional vs. sham: t_[10]_ = 2.20, p = 0.12). Within both photothrombotic groups, peri-infarct vascular density was significantly greater than that of contralesional cortex (targeted: 100–500 µm, t_[7]_ = 2.36, p = 0.0002; 500–900 µm, t_[7]_ = 2.36, p = 0.0007; traditional: 100–500 µm, t_[7]_ = 2.36, p = 0.001; 500–900 µm, t_[7]_ = 2.36, p = 0.02). These data suggest a similar magnitude of neovascularization in peri-infarct cortex 1–2 weeks after both types of photothrombosis.

### Artery-targeted photothrombotic infarcts in MC impaired forelimb function in mice

Several previous studies have found that traditional photothrombotic infarcts of MC impair skilled reaching behavior of the contralateral forelimb^40–50^. We examined the impact of artery-targeted photothrombosis to MC on forelimb function and found that photothrombotic infarcts significantly impaired performance on a skilled reaching task compared to sham operates (Fig. 5c and Suppl. Fig. 1). Repeated measures ANOVA of post-operative reaching performance revealed a significant main effect of Group (Infarct vs. Sham, F_[1,23]_ = 145.50, p < 0.0001), but not Group by Day interaction (F_[3,69]_ = 2.77, p = 0.06). However, by day 20 performance levels were similar between the infarct and sham group. These data support that artery-targeted photothrombosis is suitable for creating focal and reproducible lesions to MC that can be used to model forelimb impairments.

## Discussion

The traditional photothrombotic stroke model is strong for modeling impairments and studying mechanisms of recovery following ischemia. However, it creates a relatively thin penumbra, making it challenging to understand how events within it impact cellular mechanisms of recovery^57^. In the present study, we demonstrated that confining illumination to select cortical surface arteries increased the size of the penumbra, as evidenced by the presence of graded CBF deficits encompassing a broader cortical area compared with the traditional approach. Group differences in CBF deficits at 48 h at the furthest distance from the core became more pronounced at 120 h, at which point the targeted group showed greater CBF deficits compared to the traditional group regardless of distance.

Several other variations of photothrombosis, including the photothrombotic-ring model^58^, and single-point occlusion model^59–61^, have been demonstrated to produce a larger vascular penumbra. In the photothrombotic-ring model, the penumbral zone is contained within the ring-shaped core. Therefore, progressive vasogenic edema emanating from the ischemic ring results in inevitable deterioration of the penumbral region, limiting the examination of tissue recovery over longer periods.

In the single-point occlusion model, illumination through a two-photon microscope generates spatially restricted photoactivation of vessels confined to a diameter smaller than the targeted artery (~20 µm), resulting in relatively small ischemic lesions, and making it challenging to apply to more than one vessel at a time^59–61^. Here, we demonstrated that artery-targeted photothrombosis can occlude multiple branches of the MCA, as well as limit collateral CBF by simultaneously occluding branches of the anterior cerebral artery (ACA), thus giving the experimenter better control over reperfusion.

Photothrombosis has also been adapted for distal MCA occlusion (MCAo), resulting in more reproducible infarcts in the extent of tissue damage compared to other MCAo models, while maintaining a vascular penumbra^62,63^. Illumination with an infrared laser can be used to stimulate recanalization, such that this model can also serve as an ischemia-reperfusion model. However, infrared laser illumination triggers immediate restoration of CBF to baseline levels, whereas in clinical strokes spontaneous reperfusion can unfold very gradually and is often incomplete^64^. Our finding that reperfusion of targeted arteries was quite delayed (120 h) supports that reperfusion in the present model unfolds slowly, similar to what is seen in humans. This is also in contrast to the traditional photothrombotic model, which often results in permanent occlusion of surface arteries and relatively limited collateral flow^23,25^, and supports that it could serve as a delayed ischemia-reperfusion model, a topic of current therapeutic interest^5,9^. However, a more detailed analysis of the precise time course and assessment of boundary conditions of reperfusion are needed to understand the new model’s potential as a delayed reperfusion model.

In other rodent models of focal ischemia, increased vascular density has been linked to the infiltration of macrophages that clear necrotic tissue around the lesion site^31,35^ and to improved functional outcomes at longer time periods^12–18^. We found that vascular density was similarly increased in both photothrombotic models in the first 2 weeks post-infarct. To the extent that new vessel densities reflect neovascularization, these results support that both models instigated a similar magnitude of neovascularization despite differences in CBF deficits in the peri-lesion cortex.

Photothrombotic infarcts have been a popular choice for the purpose of modeling upper-extremity impairments^40–50^. In the present study we demonstrated that artery-targeted photothrombosis maintained the ability to create focal infarcts to the forelimb representation region of motor cortex to result in deficits in forelimb motor function. However, these deficits were no longer evident at the last endpoint, a transience that is likely attributable to the fact that the infarcts were relatively small. Preliminary results from a subsequent study indicate more persistent skilled reaching impairments after larger infarcts that were achieved by greater constraint of collateral flow to the forelimb region (i.e., by simultaneously targeting anterior, in addition to middle, cerebral artery branches)^65^.

In the present study we found that confining laser illumination to individual arteries on the cortical surface with a digital micromirror device expanded the ischemic penumbra, supporting its usefulness for examining the impact of remodeling events within the penumbra on mechanisms of recovery from ischemia. In addition, artery-targeted photothrombosis maintained the strengths of the traditional model, in that it created focal and reproducible infarcts to motor cortex that impaired forelimb motor function, making it also suitable for modeling post-stroke upper-extremity impairments Table 1.

**Table 1:** Experimental Subject Numbers.

*CBF and Vascular Study*	CBF		Vascular Density	
	*(n* = *18)*		*(n* = *20)*	
*Group*	Male	Female	Male	Female
Traditional	4	2	3	4
Targeted	4	4	5	4
Sham Young-Adult	2	2	2	2
Total	10	8	10	10
***Behavioral Study (n*** **=** ***23)***				
***Group***	**Male**	**Female**		
Infarct	7	6		
Sham	6	4		
Total	13	10		

Note. N’s do not include animals omitted from the study based on criterion defined prior to experimental procedures or due to complications, which are reported in Materials and Methods.

## Supplementary information

Supplementary Materials

## Data Availability

The datasets generated during and/or analyzed during the current study are available from the corresponding author on reasonable request.
