# Supplementary material for: Artery targeted photothrombosis widens the vascular penumbra, instigates peri-infarct neovascularization and models forelimb impairments
Source: Sci Rep. 2019 Feb 20;9:2323. doi: 10.1038/s41598-019-39092-7 (PMC6382883; doi:10.1038/s41598-019-39092-7)
Supplement: Supplementary file 1 — Supplementary Materials [file 41598_2019_39092_MOESM1_ESM.docx]

**Artery targeted photothrombosis widens the vascular penumbra, instigates peri-infarct neovascularization and models forelimb impairments.**

Dr. Taylor A. Clark^1^, Colin Sullender^2^, Dr. Shams M. Kazmi^2^, Brittany L. Speetles^3^, Michael R. Williamson^1^, Daniella M. Palmberg^4^, Dr. Andrew K. Dunn, and Dr. Theresa A. Jones^1^

**Supplementary MaterialsSupplementary Age Comparison**

**Methods**

A separate cohort of middle-aged (10-12 mo; *n* = 5 male, *n* = 5 female) mice receiving artery-targeted photothrombosis were compared with the young adult group of the primary article with the same infarct condition to examine age effects on the patterns of CBF and vascular density changes. Not included in the middle-aged n’s above were 4 animals that were omitted due to lesions that were much larger than the average (1 male and 1 female), issues with window clarity (1 male), or early post-operative death (1 male).

**Results**

*CBF responses to artery-targeted photothrombosis were similar between age groups*

Post-ischemic CBF patterns were not significantly different between young-adult and middle-aged groups (Suppl. Fig. 1). ANOVAs revealed no significant effect of age (6h: F_[1, 10]_ = 0.27, p = 0.60; 48h: F_[1, 13]_ = 3.0, p = 0.10; 120h: F_[1, 12]_ = 1.18, p = 0.30) or Age by Distance interactions at any time point (6h: F_[3, 30]_ = 0.83, p = 0.44; 48h: F_[3,39]_ = 2.04, p = 0.15; 120h: F_[3,36]_ = 1.16, p = 0.45). The tendency for CBF to be higher in middle-aged animals >500 µm from the ischemic core at 48 and 120 h was not significant (Suppl. Fig. 1B). Thus, at least by middle age, there was not a significant age effect on cortical CBF responses to artery-targeted photothrombosis.
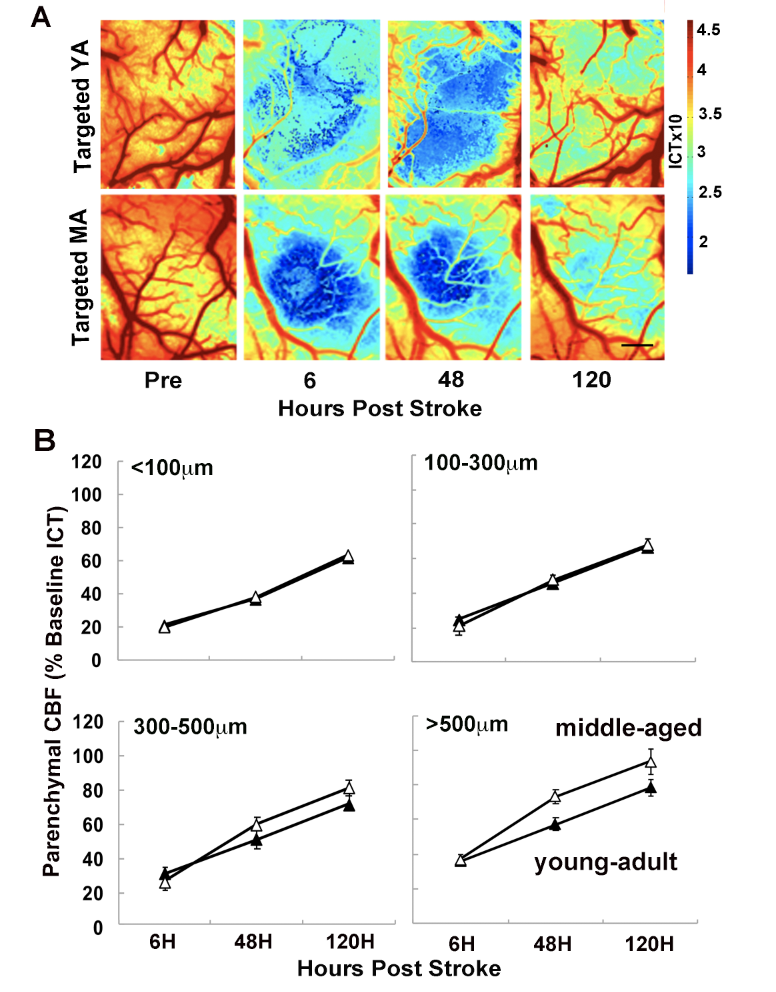


**Supplementary Figure 1**. **(A).** Parenchymal CBF patterns in the targeted middle-aged group were not significantly different from the young-adult group. **(B).** Arterial CBF patterns were not significantly different between groups. Data from the young-adult group are the same as shown in Fig. 2 of the article.

*Lesion volume, but not vascular density, significantly varied with age after artery-targeted photothrombosis*

Age-dependencies in cortical damage and vascular structural patterns were also assessed (Suppl. Fig. 2). Although lesion volumes were significantly larger in the middle-aged compared to young-adult animals (t_[12]_ = 2.17, p = 0.014 Suppl. Fig. 2A,B), consistent with several previous findings of increased infarct size with aging^1-3^ we found no effect of age on vascular density. The density of IB4+ vessels in peri-infarct cortex was not different between young-adult and middle-aged groups at either distance (100-500 µm, t_[12]_ = 2.18, p = 0.14; 500-900 µm, t_[12]_ = 2.18, p = 0.29). However, area fractions of IB4+ vessels in the contralateral cortex were significantly reduced in the middle-aged compared to young-adult group (t_[12]_ = 2.17, p = 0.02; Suppl. Fig. 2C) potentially reflecting age-related declines in vascular density. Contralateral cortical volumes were similar between targeted middle-aged and young-adult groups (middle-aged: 57.2 ± 3.18; young-adult: 61.7 ± 4.8; t_[12]_ = 2.17, p = .15). As with young adults, there was significantly greater vascular density in peri-infarct compared with contralesional cortex at both distances from the infarct border of the middle-aged group (100-500 µm: t_[5]_ = 2.57, p = 0.0001; 500-900 µm: t_[5]_ = 2.57, p = 0.001). Together, these results suggest that the vascular structural response to targeted photothrombosis was similar between age groups. Across age groups there was no significant correlation between lesion size and vascular density (100-500 µm: r = 0.12, p = 0.67; 500-900 µm: r = -0.10, p = 0.97).


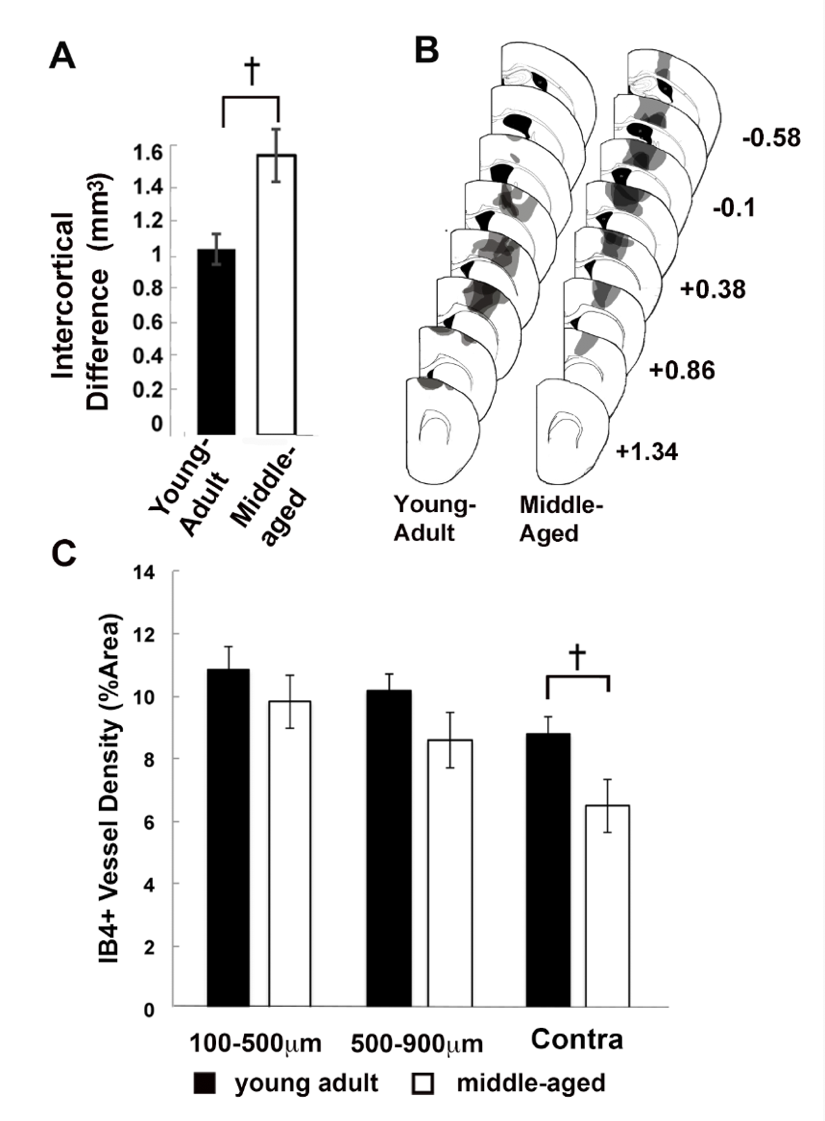


**Supplementary Figure 2**. **(A)** Infarct sizes, as estimated by the difference between contralesional and peri-infarct cortical volumes, were significantly larger in the targeted middle-aged group compared to the young adult group. †p = 0.01. **(B).** Representative lesion reconstructions of each infarct group overplayed on coronal section templates. Numbers to the right are anterior to posterior coordinates (mm) relative to bregma. **(C).** Area fractions of vessels in the ipsilesional cortex were similar between young-adult and middle-aged animals between 100-500 µm and 500-900 µm**.** Area fractions in the contralateral cortex were significantly reduced in middle-aged compared to young-adult animals. †p < 0.02, Targeted MA versus Targeted YA. Data from the young-adult group are the same as shown in Figs. 3 and 4 of the article.

**Summary of Age-Comparison Results**

We found that targeted-artery photothrombosis instigated similar CBF and vascular structural responses in middle-aged animals compared with young adults. We also found that the middle-aged group had significantly reduced vessel density in contralateral cortex compared to young adults. However, because a middle-aged sham group was not included, we cannot determine whether these results might reflect an age-associated decrease in vascular density, an age-dependency in remote vascular structural responses to ischemia, or a combination of the two. Middle-aged animals did have significantly larger lesion volumes, but this was not related to differences in vascular density relative to young adults. Together, these results suggest that, while middle-age is potentially associated with some decline in vascular density, the neovascularization response to ischemia remains robust.

**
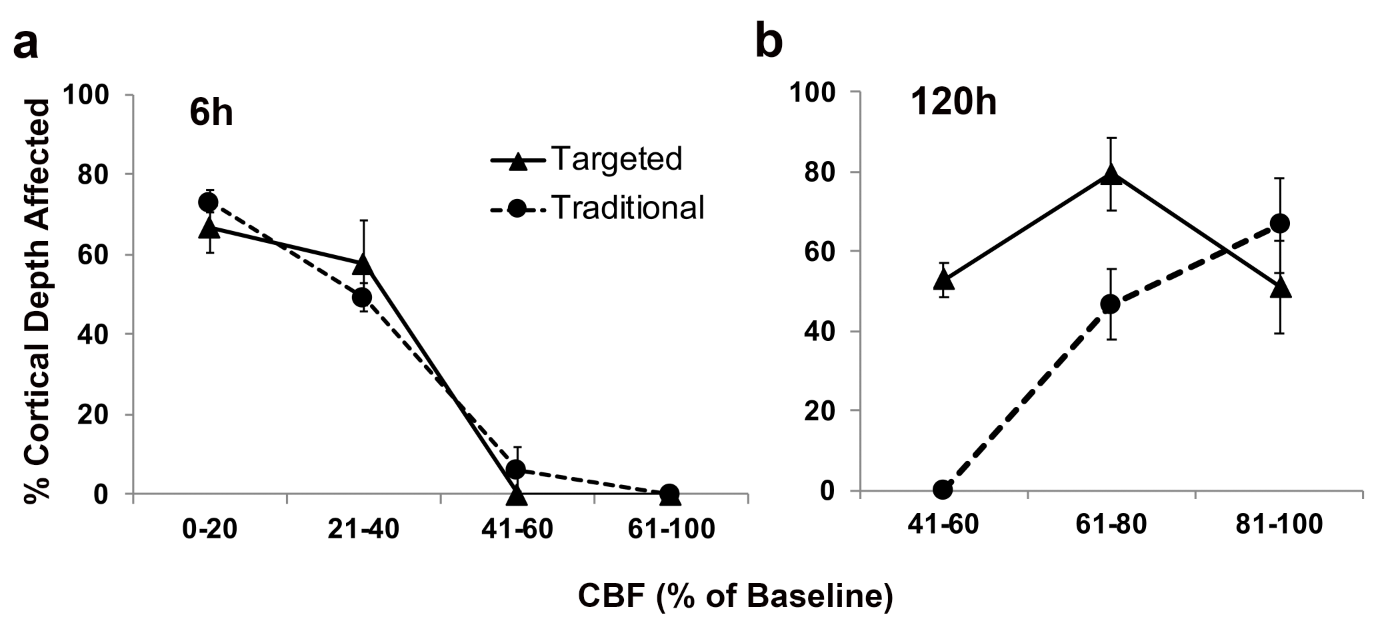
**

**Supplementary Figure 3.** Relationships between cortical damage (lesion depth) and CBF deficits assessed with MESI at **(A)** 6 h and **(B)** 120 h. At 6 h**,** the greatest cortical damage corresponded to areas where CBF fell to or below 20% of baseline CBF. The pattern was similar to the 48 h time point, but with a less steep reduction in cortical depth affected moving from regions in which CBF was below 20% to those in which it was 21-40% of baseline levels (Fig. 3). Areas where CBF was at or above 40% of baseline CBF at 6 h showed little to no cortical damage in histological sections. At 120 h, there was little to no remaining tissue with CBF reductions below 40% of baseline in mice across conditions and there was no longer a positive relationship between magnitudes of histological damage and CBF reductions. The variation in the pattern between groups reflects that the targeted group had greater region of persisting CBF reductions at 120h but had a similar extent of cortical damage as the traditional group.

| **Supplementary Table 1. CBF disaggregated by sex at each imaging time point** | | | | | | | |  |
| --- | --- | --- | --- | --- | --- | --- | --- | --- |
|  | | | | | | | |  |
| **Parenchymal CBF** | | | | | | | |  |
| **6h < 500** µ**m > 500** µ**m** | | | | | | | |  |
| *Group* | | **Female (n)** | | **Male (n)** | | **Female (n)** | **Male (n)** | |
| **Sham** | 96.2 ± 1.0 (2) | | 96.3 ± 4.5 (2) | | 96.2 ± 1.0 (2) | | 1 00 ± 1.9 (2) | |
| **Traditional YA** | 31.3 ± 3.7 (3) | | 23.0 ± 4.0 (3) | | 32.0 ± 5.1 (3) | | 49.4 ± 3.4 (3) | |
| **Targeted YA** | 18.0 ± 7.7 (2) | | 25.6 ± 3.1 (2) | | 23.0 ± 0.6 (2) | | 35.8 ± 3.4 (2) | |
| **Targeted MA** | 23.8 ± 2.9 (3) | | 21.7 ± 4.9 (2) | | 32.1 ± 7.7 (3) 42.1 ± 3.0 (2) | | | |

| **48h < 500** µ**m > 500** µ**m** | | | | | | | | | | | | | | |  |  |  |  |  |  |  |
| --- | --- | --- | --- | --- | --- | --- | --- | --- | --- | --- | --- | --- | --- | --- | --- | --- | --- | --- | --- | --- | --- |
| *Group* | | **Female (n)** | | | **Male (n)** | | | | **Female (n)** | | | | **Male (n)** | | | | |  |  |  |  |
| **Sham** | | 100.± 5.0 (2) | | | | 101 ± 0.6 (2) | | 97.4 ± 0.4 (2) | | | | 1 05 ± 0.3 (2) | | | | | | | |  |  |
| **Traditional YA** | | 49.0 ± 7.9 (3) | | | | 44.0 ± 4.6 (3) | | 85.7 ± 4.6 (3) | | | | 79.3 ± 2.0 (3) | | | | | | | |  |  |
| **Targeted YA** | | 44.4 ± 2.9 (4) | | | | 45.4 ± 4.9 (4) | | 52.6 ± 3.3 (4) | | | | 61.8 ± 6.6 (4) | | | | | | | |  |  |
| **Targeted MA** | | 49.6 ± 4.9 (3) | | | | 44.5 ± 4.3 (2) | | 78.6 ± 7.7 (3) 9 0.1 ± 4.9 (2) | | | | | | | | | | | |  |  |
| **120h < 500** µ**m > 500** µ**m** | | | | | | | | | | | | | | | | |  |  |  |  |  |
| Group **Female (n) Male (n) Female (n) Male (n)** | | | | | | | | | | | | | | | | | | | |  | |
| **Sham** | | 92.9 ± 4.0 (2) | | | | 101 ± 0.8 (2) | | | | | 96.8 ± 2.9 (2) | | | 102 ± 1.2 (2) | | | | |  |  |  |
| **Traditional YA** | | 88.9 ± 7.2 (3) | | | | 81.1 ± 1.6 (2) | | | | | 85.7 ± 4.6 (3) | | | | 89.3 ± 2.0 (2) | | | | | |  |
| **Targeted YA** | | 61.4 ± 7.2 (4) | | | | 72.8 ± 7.8 (4) | | | | | 52.6 ± 3.3 (4) | | | | 61.8 ± 6.6 (4) | | | | | |  |
| **Targeted MA** | | 77.6 ± 8.0 (3) | | | | 64.7 ± 8.9 (2) | | | | | 78.6 ± 7.7 (3) | | | | 64.7 ± 8.8 (2) | | | | | |  |
|  | | |  |  | | |  | | |  | | | | | | | | | | | |

***Arterial CBF*** ***6h 48h 120h*** *k*

| *Group* | **Female** | **Male** | **Female** | **Male Female Male** |  |
| --- | --- | --- | --- | --- | --- |
| **Sham** | 95.3 ± .1 | 101 ± .7 | 96.3 ± 2.1 | 97.8 ± 2.3 96.7 ± 0.9 97.8 ± 2.3 |  |
| **Traditional YA** | 18.8 ± 3.2 | 15.4 ± 1.3 | 27.7 ± 7.6 | 26.6 ± 2.0 59.3 ± 4.2 59.2 ± 21.8 |  |
| **Targeted YA** | 15.2 ± 1.4 | 18.6 ± 1.1 | 40.2 ± 5.3 | 49.8 ± 9.6 91.7 ± 9.2 89.3 ± 3.2 |  |
| **Targeted MA** | 13.6 ± 3.7 | 22.2 ± 8.2 | 39.3 ± 10.4 | 41.3 ±6.6 75.5 ± 21.9 94.7 ± 24.3 |  |

*Note.* Values are *M* ± *SE.* Group n's for arterial CBF are the same as those of the parenchymal CBF at the same time point. CBF, cerebral blood flow.

**Supplementary Table 2. Cortical lesion volume disaggregated by sex.**

| ***CBF and Vascular Study*** |  | |  |
| --- | --- | --- | --- |
| *Group* | **Male (n)** | **Female (n)** | |
| **Traditional YA** | 4.1 ± 0.2 (4) | 2.5 ± 0.2 (4) | |
| **Targeted YA** | 3.0 ± 0.3 (5) | 4.1 ± 0.4 (4) | |
| **Targeted MA** | 5.1 ± 1.8 (2) | 5.9 ± 0.4 (4) |  |

| ***Behavioral Study*** |  |  | | |  |
| --- | --- | --- | --- | --- | --- |
| *Group* | **Male (n)** | **Female (n)** | |  |  |
| **Infarct** | 3.0 ± 0.7 (7) | 2.8 ± 0.4 (6) |  |  |  |
| **Sham** | 0.6 ± 0.8 (6) | -0.1 ± 2.0 (4) | | |  |
| *Note.* Values are *M ± SE* contralesional-ipsilesional difference in  cortical volume. | | | | | |

**Supplementary Table 3. Area fractions of IB4-labeled blood vessels in ipsilesional and contralateral homotopic cortex disaggregated by sex**

|  | | Ipsi | | | ***Contra*** | | |
| --- | --- | --- | --- | --- | --- | --- | --- |
| *Group* (n) | *100-500µm* | | *500-900µm* | | |  |  |
| **Trad YA** |  | | |  | |  |  |
| Female (4) | 12.0 ± 1.0 | | 9.8 ± 1.0 | | | 8.3 ± 0.6 |  |
| Male (4) | 10.8 ± 1.3 | | 10.9 ± 2.1 | | | 8.7 ± 0.9 |  |
| **Target YA** |  | |  | | |  |  |
| Female (4) | 10.5 ± 0.7 | | 10.4 ± 0.5 | | | 8.8 ± 0.2 |  |
| Male (4) | 11.1 ± 1.2 | | 10.7 ± 0.9 | | | 9.0 ± 1.1 |  |
| **Target MA** |  | |  | | |  |  |
| Female (4) | 9.4 ± 1.0 | | 8.1 ± 2.4 | | | 5.9 ± 1.0 |  |
| Male (2) | 10.9 ± 2.6 | | 10.8 ± 1.3 | | | 7.9 ± 1.8 |  |
| **Sham YA** |  | |  | | |  |  |
| Female (2) | 8.4 ± 0.6 | | 8.1 ± 0.5 | | | 7.8 ± 0.1 |  |
| Male (2) | 7.6 ± 0.1 | | 6.8 ± 0.05 | | | 7.4 ± 0.3 |  |

*Note.* Values are *M* ± *SE.* The contra data were pooled across the 100-500 and 500-900 µm distances. Trad, traditional photothrombotic, Target, targeted photothromobtic, YA, young adult, MA, middle aged.

| **Supplementary Table 4. Reaching performance disaggregated by sex** | | | | | | | | | |  |
| --- | --- | --- | --- | --- | --- | --- | --- | --- | --- | --- |
| **Group Pre-op (Days 10-11) Post-op Day 3 Day 20** | | | | | | |  |  |  |  |
| **Sham** | |  | |  | |  | |  |  |  |
| Female (4) | 0.35 ± 0.06 | | 0.55 ± 1.0 | | 0.38 ± 0.06 | | | |  | |
| Male (6) | 0.39 ± 0.02 | | 0.33 ± 0.04 | | 0.29 ± 0.04 | | | |  | |
| **Stroke** |  | |  | |  | | | |  | |
| Female (6) | 0.50 ± 0.03 | | 0.13 ± 0.02 | | 0.32 ± 0.02 | | | | | |
| Male (7) | 0.33 ± 0.03 | | 0.16 ± 0.02 | | 0.22 ± 0.04 | | | | | |

*Note:* Data are *M* ± *SE* number of successes per reach attempt. Group n’s are in parentheses.
